# Acute versus chronic supplementation of sodium citrate on 200 m performance in adolescent swimmers

**DOI:** 10.1186/1550-2783-11-26

**Published:** 2014-06-12

**Authors:** Colin Russell, Efthymios Papadopoulos, Yasmeen Mezil, Greg D Wells, Michael J Plyley, Mathew Greenway, Panagiota Klentrou

**Affiliations:** 1 Department of Kinesiology, Faculty of Applied Health Sciences, Brock University, 500 Glenridge Av., St. Catharines, Ontario L2S 3A1, Canada; 2 Faculty of Kinesiology and Physical Education, University of Toronto, 55 Harbord St., Toronto, Ontario M5S 2W6, Canada; 3 Michael G. DeGroote School of Medicine, McMaster University, 500 Glenridge Av., St. Catharines, Ontario L2S 3A1, Canada

**Keywords:** Ergogenic aid, Buffer, Alkalization, Responders, Blood lactate, Bicarbonate

## Abstract

**Background:**

A double-blinded, placebo-controlled, cross-over design was used to investigate whether two different sodium citrate dihydrate (Na-CIT) supplementation protocols improve 200 m swimming performance in adolescent swimmers.

**Methods:**

Ten, male swimmers (14.9 ± 0.4 years of age; 63.5 ± 4 kg) performed four 200 m time trials with the following treatments: acute (ACU) supplementation (0.5 g kg^-1^ administered 120 min pre-trial), acute placebo (PLC-A), chronic (CHR) supplementation (0.1 g∙kg^-1^ for three days and 0.3 g kg^-1^ on the forth day 120 min pre-trial), and chronic placebo (PLC-C). The order of the trials was randomized, with at least a six-day wash-out period between trials. Blood samples were collected by finger prick pre-ingestion, 100 min post-ingestion, and 3 min post-trial. Performance time, rate of perceived exertion, pH, base excess, bicarbonate and lactate concentration were measured.

**Results:**

Post-ingestion bicarbonate and base excess were higher (*P* < 0.05) in both the ACU and CHR trials compared to placebo showing adequate pre-exercise alkalosis. However, performance time, rate of perceived exertion as well as post-trial pH and lactate concentration were not significantly different between trials. Further analysis revealed that five swimmers, identified as responders, improved their performance time by 1.03% (*P* < 0.05) and attained higher post-trial lactate concentrations in the ACU versus PLC-A trial (*P* < 0.05). They also had significantly higher post-trial lactate concentrations compared to the non-responders in the ACU and CHR trials.

**Conclusions:**

Acute supplementation of Na-CIT prior to 200 m swimming performance led to a modest time improvement and higher blood lactate concentrations in only half of the swimmers while the chronic Na-CIT supplementation did not provide any ergogenic effect in this group of adolescent swimmers.

**Trial registration:**

Clinicaltrials.gov NCT01835912.

## Introduction

Alkalizing agents have been used in high performance sports as a strategy to postpone the onset of fatigue during high intensity exercise by slowing the decline in muscle and blood pH [1,2]. Studies have confirmed that increasing the extracellular pH, via an alkalizer, promotes the efflux of lactate and H^+^ from the active muscles [1,3-5]. Therefore, artificially inducing alkalosis prior to anaerobic exercise may reduce intracellular acidosis and increase the time to fatigue [6,7]

The process known as “bicarbonate loading”, in which sodium bicarbonate is ingested pre-performance, is a popular method of blood alkalization among athletes [6,8]. According to a recent meta-analysis by Carr et al. [8], sodium bicarbonate enhances performance by 1.7% (±2.0%) for a 60 sec maximal effort, with a dose of 0.3 g kg^-1^ of body mass being the optimal dose. However, the gastrointestinal (GI) acceptance profile of sodium bicarbonate is narrow and 10% of humans cannot adequately tolerate the doses needed to elicit an ergogenic effect [6,9]. Thus, ingesting sodium bicarbonate in high enough doses to induce an adequate modification of the acid–base balance during exercise can be detrimental to performance [6,9,10]. Sodium citrate (Na-CIT) is another alkalizing agent that has been studied in sports over a broad array of doses, times and distances but the results on its ergogenic effect have been inconclusive [2-4,10-14]. Indeed, the meta-analysis by Carr et al. [8] reported an unclear effect on performance (0.0 ± 1.3%) for a 60 sec maximal effort, with a dose of 0.5 g kg^-1^. Due to this uncertainty, in combination with its lower commercial availability, Na-CIT has not been used as an alternative to sodium bicarbonate although it has a higher GI tolerance [2,5,6]. Na-CIT can enter the sarcolemma through a recently discovered plasma membrane citrate transporter [15], providing new evidence to support its potential effect on performance.

Competitive swimming is an ideal model for studying the effectiveness of alkalizing agents due to its high reliance on anaerobic metabolism. Events range in duration from 22 sec (50 m freestyle) to 15 min (1500 m freestyle) with the highest blood lactate concentrations found in the 200 m (~2 min) events. Typical post-race blood lactate concentrations for these events are 6.4, 9.1, and 14.0 mmol/L in the 1500 m, 50 m, and 200 m events, respectively [16]. Furthermore, swimmers often compete in several events within a 30–90 min time frame during any given session. Swimmers must also contend with restrictions placed on their breathing frequency during intense exercise as a result a unique interaction between muscle physiology, technique, and ventilation. Exercise hyperpnoea is limited during high intensity swimming because turning or lifting the head to breathe may jeopardize execution of proper stroke technique [17,18]. Indeed, swimming requires that the athlete sustain a high rate of energy expenditure and the suspension of breathing for approximately 20 - 30% of a race [19]. Given these limitations and the physiological consequences, it is likely that anaerobic metabolism is a significant contributor to metabolic power in competitive swimming, and may also be a primary determinant of fatigue and limitations in performance [7].

Another reason why competitive swimming is an appropriate model for studying the effectiveness of alkalizing agents is that swimmers are often young when they reach elite level competition; among the swimming medalists in the 2012 Olympics (*n* = 78), twenty-five were under 21 and eight were under 18 years old. This creates a highly competitive environment, where 80% of elite adolescent athletes are using supplements and other non-doping strategies to improve performance [20]. It is, therefore, surprising that there is such a lack of research on the effectiveness of such ergogenic aids in this population [20], especially when acid base regulation in adolescents may be significantly different than that of adults.

The overall purpose of this study was to evaluate the ergogenic effect of two Na-CIT supplementation protocols, previously used in adults, in adolescent swimmers. Specifically, the types of Na-CIT supplementation protocols that have been previously applied include an acute (single) dose and a chronic (multi-day) dose prior to performance. During the acute delivery mode participants take one single dose (0.3 - 0.6 g∙ kg^-1^ body mass Na-CIT) 60 to 180 min before the start of competition [2-4,11,13] while a chronic dose (0.3 g∙ kg^-1^ body mass Na-CIT) is given for a number of days prior to performance [21]. Chronic dosing of alkalizing agents was first employed by McNaughton et al. [22] using sodium bicarbonate in an effort to elicit an ergogenic effect while minimizing GI upset, which often occurs with acute dosing protocols. Based on these studies, a double-blinded, placebo controlled, cross-over design was used to investigate the effects of an acute versus a chronic Na-CIT supplementation protocol on 200 m swimming performance and acid–base parameters in male, adolescent swimmers.

## Methods

### Participants

Sample size was calculated using pre- and post-trial blood lactate concentrations from a published 5 km run trial in adults, an 80% power, and a 0.05 level of significance; this resulted in a minimum sample size of 8 [13]. Thus, ten, well trained, male, adolescent swimmers (14.9 ± 0.4 years of age; 63.5 ± 4.0 kg), were recruited from competitive swimming clubs in Ontario, Canada to participate in the current study. All participants had at least 3 years experience in competitive swimming and had achieved regional, provincial and/or national level qualifications. Informed consent was obtained from all participants and their parents. The study procedures were approved by the Health Canada Natural Health Products Directorate and the Brock University Research Ethics Board.

### Experimental design

The current study used a randomized, double-blinded, placebo controlled, cross-over design. All participants performed four swimming trials under four treatment conditions determined by the amount of, and time over which, Na-CIT dihydrate [(HCOONa)_2 *_ 2(H_2_O)] was ingested. Specifically, all participants randomly performed the following 4 trials; two experimental: 1) acute (ACU), 2) chronic (CHR), and 2 placebo: 3) acute placebo (PLC-A), and 4) chronic placebo (PLC-C). Each Na-CIT supplementation trial was separated by at least a six-day washout period. The order of trials was randomly assigned to each participant by a computerized random number generator.

The study was conducted during the mid-season training period (March-April) in a 4-week window without competition in order to minimize fluctuations in training volume and tapering effects. During this period, the swimmers trained 14–19 hours/week including 12–16 hours of swimming sets and 0–5 hours of weight training. Their training consisted of: a) seven to nine variant-load swimming sessions per week of medium to high-intensity, and b) two to three constant-load weight training sessions per week. The participants were instructed to maintain their individual training programs. Additionally, they were advised to refrain from any high-intensity exercise and to continue their nutritional habits between the four swimming trials.

### Supplementation protocol

Sodium Citrate (Victoria Compounding Pharmacy) was delivered in solution with 500 mL of flavored water (Crystal Light Pink Lemonade); the placebo consisted of similarly flavored water (Crystal Light Pink Lemonade). Ten adult volunteers tested multiple flavors (Strawberry-Banana-Orange, Lemon-Lime, and Pink Lemonade), with and without Na-CIT, to find an optimal masking flavor in an effort to maintain blinded supplementation. Volunteers were blinded to which samples contained Na-CIT. After sampling and revealing which drinks contained Na-CIT, the volunteers chose the Pink Lemonade as the best masking flavor for the supplementation protocols.

According to McNaughton [4], the optimal ACU dose of Na-CIT was 0.5 g kg^-1^; therefore, the ACU trial involved taking 0.5 g kg^-1^ of Na-CIT in solution with 500 mL of flavored water consumed 120 min prior to performance according to the timing protocol described by Oopik et al. [13]. The CHR dose involved taking 0.1 g kg^-1^ of Na-CIT three times per day for three days, and on the fourth day, a final dose of 0.3 g kg^-1^ was consumed 120 min prior to performance as previously done in adult athletes [21]. The PLC-A and PLC-C involved 500 mL of flavored water taken with the same frequency and timing as their corresponding experimental trial. The doses and the ingestion time frame of 120 min pre-trial were chosen to match previously published protocols using Na-CIT supplementation [13,23]. It is recognized that there are different ingestion times suggested in the literature, anywhere from 60 to 120 min pre-performance [6,22]. However, since all previous studies are in adult athletes and this is the first exploratory pediatric study the decision was to start with the time frame previously used for Na-CIT [13,21].

The placebo and Na-CIT bottles were coded by an independent researcher, and the key was used only at the time of data analysis by the primary investigator. Swimmers were simply asked anecdotally if they knew which solution they were ingesting and if they were experiencing any GI discomfort throughout each trial. In all cases, swimmers did not know which solution they were ingesting and no GI discomfort was reported during the study.

### Swimming trials

The 200 m swimming trials were conducted in a short-course (25 m) pool. Participants swam a 200 m event of their preferred stroke at maximal effort. The choice of stroke was given to increase participant motivation and provide real life data. For each swimmer, the same stroke was used for all four trials (backstroke n = 1, breaststroke n = 2, freestyle n = 6, individual medley n = 1). The breaststrokers and three freestylers (n = 5) were National age group qualifiers, the backstroker and 2 freestylers were provincial qualifiers (n = 3), and the rest were regional qualifiers (n = 2). All swimmers wore the same, regular competition apparel across the four trials. Warm-up and warm-down procedures were based solely on each swimmer’s typical competition routine. Every trial was done during the same time of the day (5:00–6:00 pm) in order to minimize diurnal and daily variations. The 200 m swim began with a dive from the blocks with a typical competition signal by the same starter. Performance times and rates of perceived exertion (RPE) were recorded at the end of each trial. Performance times were recorded with a manual stopwatch by the same investigator.

### Blood sampling and analysis

Blood was collected pre-ingestion, 100 min post-ingestion (20 min pre-trial), and 3 min post-trial. The post-trial collection time was chosen based on previous research suggesting that blood lactate reaches its highest concentrations between 3–5 min post-exercise [16,24-26]. A mixed blood sample was collected by finger prick and analyzed immediately using an automated lactate analyzer (Arkray Lactate Pro LT-1710) to determine blood lactate concentrations. Using the same finger prick, ~95 μL blood was collected into a plain capillary tube and immediately analyzed for base excess (BE), bicarbonate, pH, and PCO_2_ using an i-STAT 1 analyzer (Abbott Point of Care). The blood collection was consistently done by the same researcher for each analyzer and for all trials.

### Statistical analysis

Sample size was calculated using pre- and post-trial blood lactate concentrations from a published 5 km run trial in adults, an 80% power, and a 0.05 level of significance; this resulted in a sample size of 8 [13]. The Statistical Package for Social Sciences (SPSS Inc., Version 19.0) was used for all data analyses, and statistical significance was accepted at *P* < 0.05. Descriptive data are presented as mean ± SEM.

Repeated measures ANOVA analysis was used to compare performance time and blood lactate concentrations among trials, and RPE to establish equal effort among all trials. Due to missing data points, BE, bicarbonate, pH, and PCO_2_ were analyzed for differences between trials using an ANOVA and the assumption of equal sample sizes was not satisfied. This was accounted for in simple comparisons using a Gabriel’s *post-hoc*. In addition, the time effects within trials for all physiological variables were analyzed using repeated measures ANOVA.

Further analysis was conducted within two sub-groups: “responders” and “non-responders”, in which the athletes were “barred” on the basis of performance differences. Participants were classified as responders if they had a performance improvement greater than 0.4% in the ACU versus the PLC-A trial. This is considered a significant competitive improvement estimated by analyzing the magnitude of the improvement needed for a swimmer ranked in the Top 10 in the World to medal in the Olympics [27,28]. Of the ten swimmers, five were identified as responders. Anthropometric data were compared between responders and non-responders for differences in age and body mass using an independent sample T-test. Due to the small sample size, the responders’ group did not satisfy the assumptions of normality for time and lactate concentrations, and therefore, were analyzed with a non-parametric Wilcoxon Signed Ranks test. Lactate concentrations of responders and non-responders were compared using a Mann–Whitney U test.

## Results

There were no differences in performance times between the PLC-A and PLC-C trials (143.5 ± 4.7 and 143.5 ± 5.4 sec, respectively), indicating that the young swimmers were able to accurately reproduce their performance. When comparing the PLC-A versus the ACU trial, the PLC-C versus the CHR trial, and the ACU versus the CHR trial for all swimmers, no significant differences were found. Furthermore, RPE was not statistically different across all trials, confirming that the perception of effort was unaffected by any perception (or absence of) in regards to the nature of the supplement.

**Figure 1:**
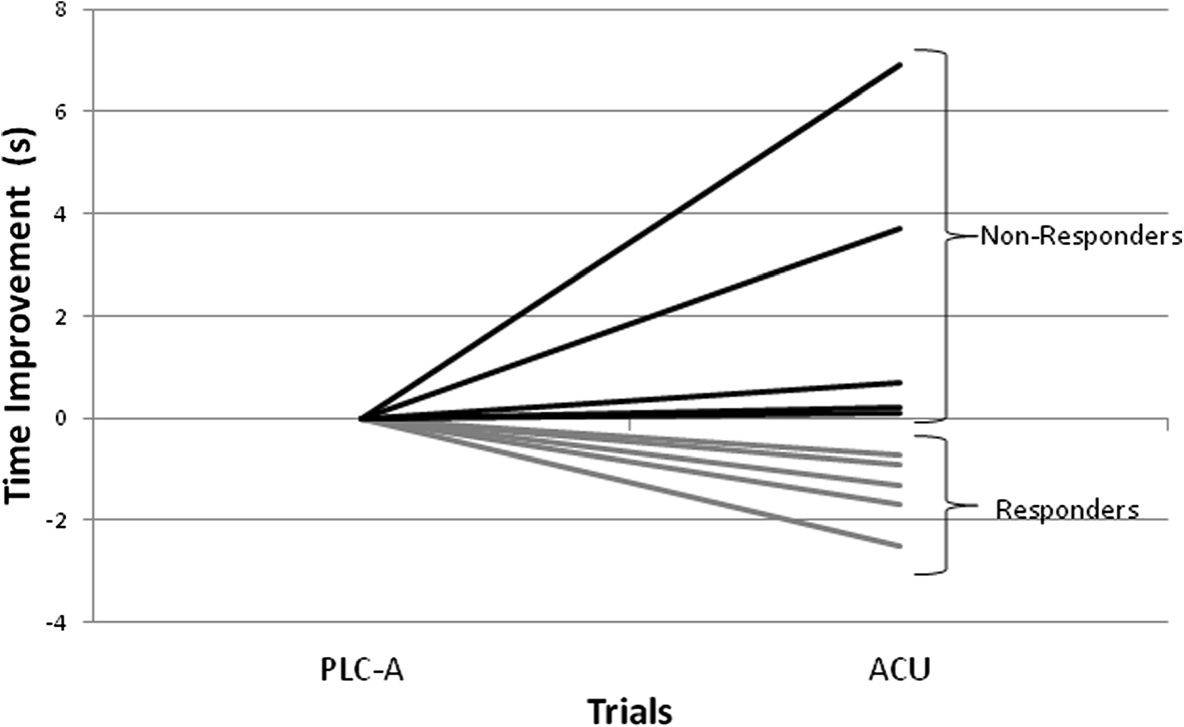
**Absolute change in performance time for the responders (n = 5) and non-responders (n = 5) comparing acute (ACU) versus acute placebo (PLC-A) supplementation trials.** Performance was significantly different in the ACU versus PLC-A (*P* < 0.05). Each *line* represents a different swimmer.

The five swimmers, identified as responders, improved their performance times by 1.03% (*P* < 0.05) in the ACU compared to the PLC-A trial (Figure 1). This improvement was statistically significant and more than the set minimum improvement of 0.4%. However, even after applying the 0.4% minimum improvement requirement there were no significant performance differences in the CHR compared to the PLC-C trial. In addition, no significant ergogenic or ergolytic effect was found in the non-responders. Although statistically non-significant, the five swimmers classified as responders were older and had a higher body mass and BMI than the non-responders (Table 1).

**Table 1:** Physical characteristics (mean ± SEM) of both the 5 responders and 5 non-responders

	**Age (yrs)**	**Body mass (kg)**	**Height (cm)**	**BMI (kg/m**^**2**^**)**
All	14.9 ± 0.4	63.5 ± 4.0	168.6 ± 8.3	21.0 ± 0.6
Responders (n = 5)	15.4 ± 0.5	67.4 ± 4.1	172.2 ± 4.7	22.1 ± 1.1
Non-Responders (n = 5)	14.4 ± 0.4	59.3 ± 3.8	163.7 ± 2.2	19.8 ± 0.6

**Figure 2:**
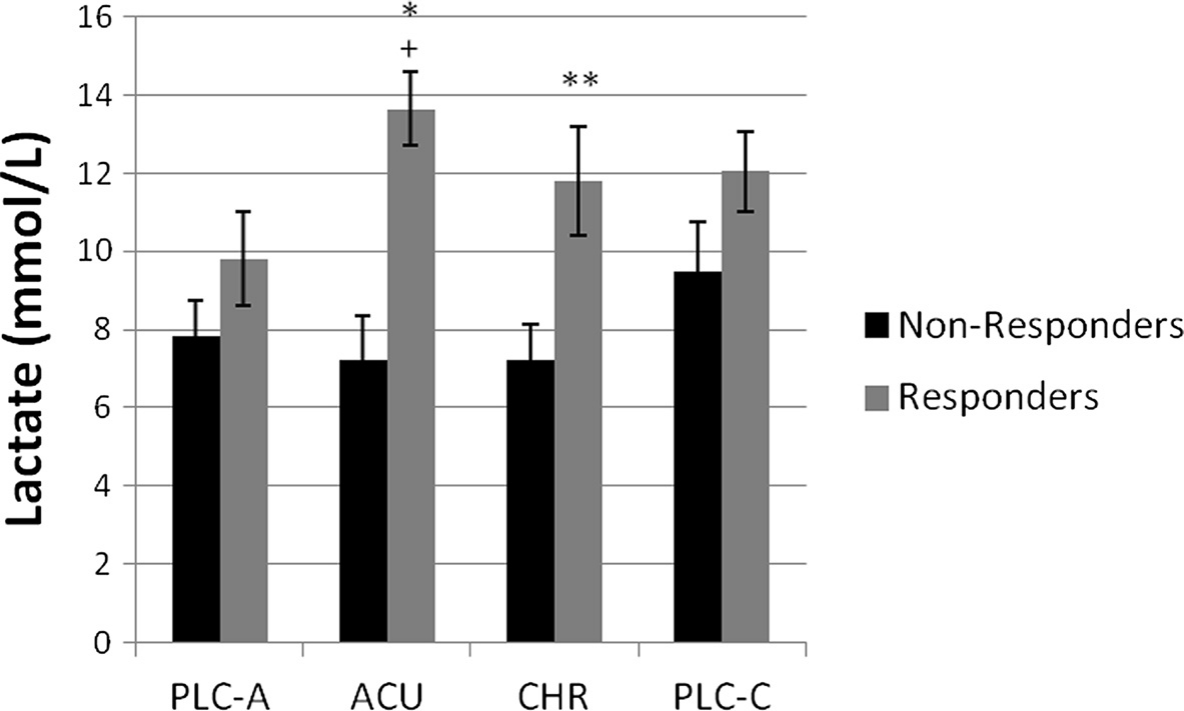
**Post-trial lactate concentrations (mmol/L) of responders and non-responders. **^a^Significantly different (*P* < 0.05) from acute placebo trial (PLC-A). ^b^Significantly different (*P* < 0.05) from non-responders in the acute (ACU) trial. ^c^Significantly different (*P* < 0.05) from non-responders in the chronic (CHR) trial. Values are Mean ± SEM.

As expected, blood lactate concentrations were significantly increased from post-ingestion to post-trial (*P* < 0.05), across all trials. The responders had significantly higher blood lactate concentrations in the ACU compared to the PLC-A trial (*P* < 0.05), but this was not the case when comparing the CHR versus the PLC-C trial. Furthermore, responders had significantly higher post-trial blood lactate concentrations than non-responders in both the ACU (*P* < 0.05) and the CHR trials (*P* < 0.05) (Figure 2).

**Figure 3:**
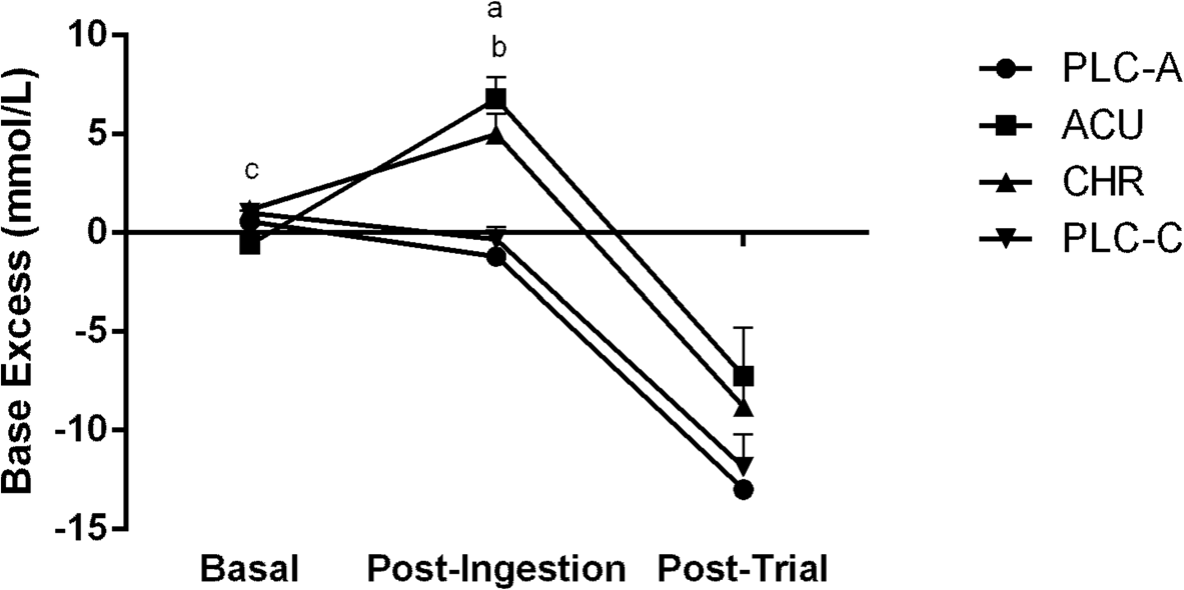
**Base excess (BE) (mmol/L) at basal, post-ingestion, and post-trial time points for the acute placebo (PLC-A), acute (ACU), chronic (CHR) and chronic placebo (PLC-C) trials. **^a^Significant difference during post-ingestion (*P* < 0.05) between ACU and PLC-A. ^b^Significant difference during post-ingestion (*P* < 0.05) between CHR and PLC-C. ^c^Significant difference during basal (*P* < 0.05) between CHR and ACU. Significant time effects (*P* < 0.05) basal to post-ingestion in ACU and PLC-C. Significant time effects (*P* < 0.05) post-ingestion to post-trial in ACU, CHR, and PLC-C. Values are Mean ± SEM.

**Figure 4:**
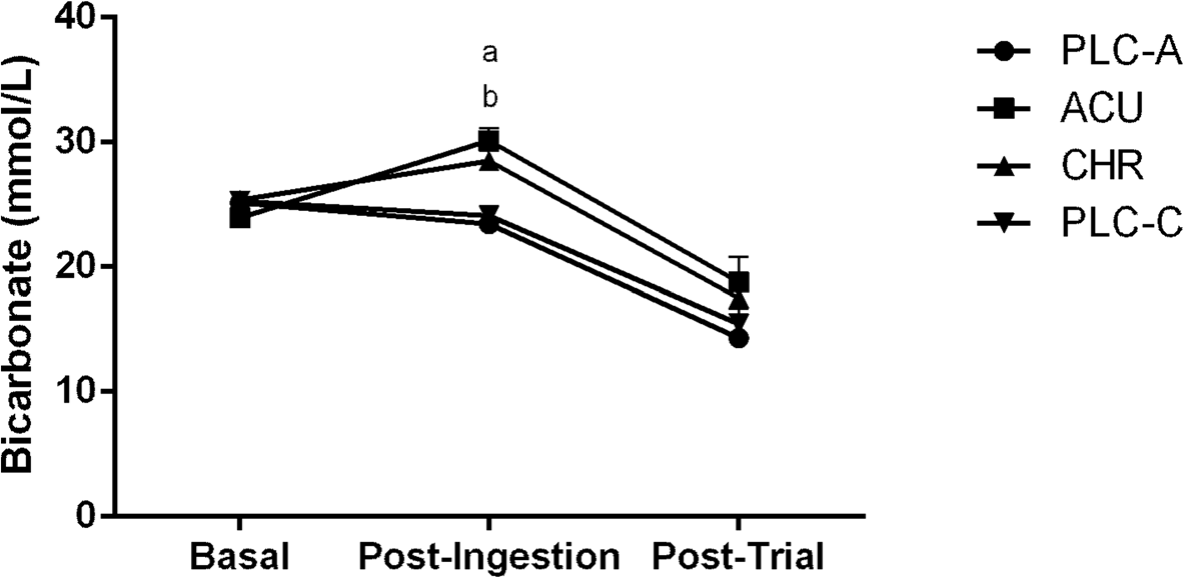
**Bicarbonate concentration (mmol/L) at basal, post-ingestion, and post-trial time points for the acute placebo (PLC-A), acute (ACU), chronic (CHR) and chronic placebo (PLC-C) trials. **^a^Significant difference during post-ingestion (*P* < 0.05) between ACU and PLC-A. ^b^Significant difference during post-ingestion (*P* < 0.05) between CHR and PLC-C. Significant time effects (*P* < 0.05) basal to post-ingestion in ACU and PLC-C. Significant time effects (*P* < 0.05) post-ingestion to post-trial in ACU, CHR, and PLC-C. Values are Mean ± SEM.

**Figure 5:**
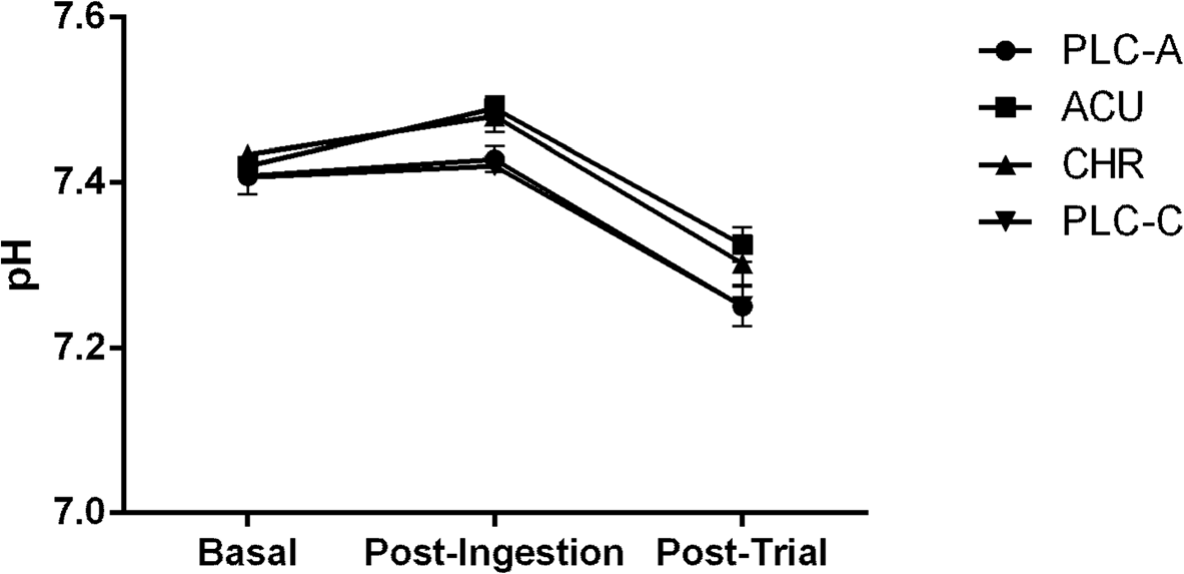
**Blood pH at basal, post-ingestion, and post-trial time points for the acute placebo (PLC-A), acute (ACU), chronic (CHR) and chronic placebo (PLC-C) trials.** Significant time effects (*P* < 0.05) from basal to post-ingestion. Trend to significance (*P* = 0.06) during post-ingestion between ACU and PLC-A. Values are Mean ± SEM.

The analysis of the time effects for BE and bicarbonate showed similar results (Figures 3 and 4). The post-ingestion values were significantly higher than the basal (*P* < 0.05) and post-trial values (*P* < 0.05). Upon further analysis, the post-ingestion values in the ACU and the CHR trials were found to be significantly higher than the basal (*P* < 0.05) and post-trial values (*P* < 0.05). As expected, pH significantly decreased from post-ingestion to post trial (*P* < 0.05); however, pH only slightly increased (*P* = 0.07) from basal to post-ingestion in the ACU trial (Figure 5). Furthermore, PCO_2_ significantly decreased from post-ingestion to post-trial (*P* < 0.05).

The between group comparisons indicated that basal BE (Figure 3) was significantly higher in the CHR trial versus the ACU trial *(P <* 0.05). Post-ingestion BE was significantly higher in the ACU versus the PLC-A trial (*P* < 0.05), and in the CHR versus the PLC-C trial (*P* < 0.05), suggesting a significant pre-exercise alkalosis in both ACU and CHR trials. However, there were no post-trial differences in BE between the Na-CIT supplementation trials and their corresponding placebo (Figure 3). As expected, post-ingestion bicarbonate concentrations were significantly different in both the ACU (*P* < 0.05) and CHR (*P* < 0.05) treatment conditions compared to their corresponding placebo (Figure 4). There was also a small, non-significant difference in the post-ingestion pH (*P =* 0.06) between the ACU and the PLC-A trial (Figure 5). However, there were no post-trial differences in bicarbonate concentration between the Na-CIT supplementation trials and their corresponding placebo. Similarly, PCO_2_ values were not significantly different between conditions.

## Discussion

This is the first study to investigate the potential ergogenic effects of Na-CIT in adolescent athletes. Ten, well-trained, adolescent swimmers performed four 200 m time trials at maximal effort, using two different Na-CIT supplementation protocols: ACU and CHR each with a corresponding placebo (PLC-A and PLC-C). The main finding was that acute supplementation of Na-CIT provided adequate pre-exercise alkalosis but did not result in an improved 200 m swimming performance or higher post-trial blood lactate concentrations in all young swimmers. This is also the first study to apply a chronic Na-CIT supplementation regimen in an effort to improve performance while minimizing GI discomfort. Indeed, the swimmers were regularly asked throughout the study if any GI discomfort occurred and none was reported. However, in our sample of adolescent swimmers, the chronic supplementation of Na-CIT did not have an effect on performance despite its significant pre-exercise alkalosis effect.

With all 10 swimmers included in the analysis, the 200 m performance times did not appear to improve with either the ACU or the CHR supplementation. Na-CIT is postulated to work predominantly as an alkalizing agent; however, more study is needed on its intracellular effects. Lactate facilitation out of working muscle is increased under alkalotic conditions compared to placebo [5]. However, post-trial lactate concentrations were also not statistically different between trials. The literature is predominantly in agreement; lactate concentrations are significantly higher post-trial with Na-CIT ingestion compared to control or placebo [4,11-14], even when performance outcomes were not improved with supplementation [2,3,29]. Therefore, a higher lactate concentration post-trial, with Na-CIT ingestion, was expected.

It is well established that energy production through anaerobic glycolysis during high-intensity exercise is lower in children than adults [30,31]. This difference has been explained by several mechanisms including reduced activity of PFK [32-35], lower activity of lactate dehydrogenase [32-35], limited ability to recruit and use type IIb motor units [34,36], and a greater reliance on aerobic oxidative enzymes [30,32,34]. Furthermore, this difference may be the reason for the smaller intramuscular pH change and lower lactate concentration found in children and adolescents after maximal exercise compared to adults [31-34,37].

Given these age related metabolic differences we further investigated the potential to find participants that responded to Na-CIT at a greater magnitude than others. Therefore, the data were analyzed for responders and non-responders. Responders were chosen if they had greater than 0.4% improvement, which corresponds to a significant competitive improvement [27,28], in the ACU versus PLC-A trials. Interestingly, the responders (n = 5) were characterized with a higher mean age and body size compared to non-responders, and had a 1.03% mean performance improvement, which was greater than expected and statistically significant, in the ACU but not in the CHR trial. The acid–base response was favorable post-ingestion amongst the responders. Similarly, post-trial lactate concentrations were significantly higher in the ACU trial as compared to its placebo, but not in the CHR trial. When compared to non-responders, responders had higher post-trial lactate concentrations in both the ACU and CHR trials. In fact, Na-CIT did not induce any ergogenic or ergolytic effect in non-responders, and they did not attain typical blood lactate concentrations after the 200 m time trials, as was observed for the responders. Therefore, those who developed higher post-trial lactate levels benefited from the acute supplementation. These findings show a more active anaerobic metabolism amongst the group of responders, which is supported by the pre-mentioned age related differences. Indeed the responders were older as a group. Furthermore, responders had greater BMI indicating a difference in body composition. It is, therefore, possible that the responders had more muscle mass potentially enhancing their use of Na-CIT, and subsequently their anaerobic metabolism.

The effect on both swimming performance and plasma alkalization was dependent on the supplementation protocol. The acute supplementation benefited the performance of the responders; however, the chronic supplementation did not lead to significant improvement or increase lactate concentration. The CHR protocol was enacted to incrementally increase plasma BE over a longer time period to allow similar blood alkalization with a smaller dose at the basal time point. The rationale behind the chronic dosing supplementation was to minimize the potential for performance inhibiting GI upset. Perhaps the CHR pre-trial dose was insufficient to elicit performance enhancement, even with the chronic dosing protocol over the previous three days. Another factor could be the time between the last chronic dose and the pre-trial dose of Na-CIT. Optimally, the pre-trial dose would have been the morning after the last chronic dose; however, the swims were performed after school, in the late afternoon. Further experimentation with the timing of the last chronic dose and the pre-trial dose may be necessary to find an optimal protocol, should one exist

Sample size was a limitation of this study as is for most studies focused on athletic enhancement of specific age groups. Considering the post-study analysis of responders and non-responders, the absence of maturation data of the participants was a limitation based on the conclusions of this study. Differences in training volume may also be a limitation to studies attempting multi-day trials over a period of time. In addition, although allowing swimmers to warm-up and race using their preferred routine and stroke was chosen to improve motivation and real-life application it is possible that the discrepancies in the warm-up routines between swimmers and the different strokes swam could have added some noise into the data that cannot be controlled. Therefore, the study cannot answer whether the degree of the observed effect (or lack thereof) was mediated, at least in part, due to the different swimming strokes and warm-up routines.

## Conclusions

This double-blinded, placebo controlled, cross-over trial of Na-CIT supplementation did not show a significant ergogenic effect in all adolescent swimmers. Specifically, acute supplementation of Na-CIT provided sufficient pre-exercise alkalosis (as shown by the higher BE and bicarbonate) for performance improvement in 200 m time trials in only half of the young swimmers, who were older and had higher body mass. Post-trial blood lactate concentrations were also higher for this group. However, the chronic supplementation protocol was not significantly advantageous for either responders or non-responders though it did provide a significantly increased BE and bicarbonate concentration post-ingestion. Adequate timing of the CHR dosing before the trial day may have been a factor in the lead up to the basal time measurement.

## Abbreviations

ACU: Acute; PLC-A: Acute placebo; BE: Base excess; CHR: Chronic; PLC-C: Chronic placebo; GI: Gastrointestinal; RPE: Rate of perceived exertion; Na-CIT: Sodium citrate.

## Competing interests

There is no conflict of interest in this study.

## Authors’ contributions

CR conceived of the study and carried out data acquisition, analysis, interpretation, and was the principal writer for the manuscript. EP participated in data acquisition and was a manuscript reviewer. YM participated in data acquisition and was a manuscript reviewer. GW conceived of the study and was a manuscript reviewer/reviser. MP carried out data interpretation and was a manuscript reviewer/reviser. MG was the medical advisor and was a manuscript reviewer/reviser. PK was the research supervisor for the study and was involved in its conception. PK also assisted in the statistical analysis and interpretation of the results, and was the senior manuscript writer/reviser. All authors read and approved the final manuscript.
